# The Integration of Eye Tracking Responses for the Measurement of Contrast Sensitivity: A Proof of Concept Study

**DOI:** 10.3389/fnins.2021.710578

**Published:** 2021-08-12

**Authors:** Yijing Zhuang, Li Gu, Jingchang Chen, Zixuan Xu, Lily Y. L. Chan, Lei Feng, Qingqing Ye, Shenglan Zhang, Jin Yuan, Jinrong Li

**Affiliations:** ^1^State Key Laboratory of Ophthalmology, Zhongshan Ophthalmic Center, Sun Yat-sen University, Guangzhou, China; ^2^School of Optometry, The Hong Kong Polytechnic University, Hong Kong, China

**Keywords:** contrast sensitivity, eye tracking, preferential-looking, psychophysical, preverbal

## Abstract

Contrast sensitivity (CS) is important when assessing functional vision. However, current techniques for assessing CS are not suitable for young children or non-verbal individuals because they require reliable, subjective perceptual reports. This study explored the feasibility of applying eye tracking technology to quantify CS as a first step toward developing a testing paradigm that will not rely on observers’ behavioral or language abilities. Using a within-subject design, 27 healthy young adults completed CS measures for three spatial frequencies with best-corrected vision and lens-induced optical blur. Monocular CS was estimated using a five-alternative, forced-choice grating detection task. Thresholds were measured using eye movement responses and conventional key-press responses. CS measured using eye movements compared well with results obtained using key-press responses [Pearson’s *r*_best–corrected_ = 0.966, *P* < 0.001]. Good test–retest variability was evident for the eye-movement-based measures (Pearson’s *r* = 0.916, *P* < 0.001) with a coefficient of repeatability of 0.377 log CS across different days. This study provides a proof of concept that eye tracking can be used to automatically record eye gaze positions and accurately quantify human spatial vision. Future work will update this paradigm by incorporating the preferential looking technique into the eye tracking methods, optimizing the CS sampling algorithm and adapting the methodology to broaden its use on infants and non-verbal individuals.

## Introduction

Early detection and treatment of vision problems in young children can prevent visual impairment and the development of amblyopia. However, quantitative measurement of visual function, especially spatial vision, in young children is complicated by their immature cognition, attention and communication.

Eye movements can be used as a non-verbal cue to determine thresholds for the detection or discrimination of visual stimuli. With the development of infrared eye tracking technology, a number of techniques for objectively estimating visual function based on eye movements have been described (Schütz et al., 2011). Specifically, the rate of microsaccades has been used to measure visual responses to visual stimuli. As such, visual acuity (VA; Adler and Fliegelman, 1934), contrast sensitivity (CS; Denniss et al., 2018), and convergence angle (Okada et al., 2006) can be estimated from microsaccade rate using an eye tracker. Optokinetic nystagmus (OKN), a series of involuntary ocular movements elicited by moving visual stimuli, has been used as visual feedback to test visual functions (Schor and Levi, 1980; Leguire et al., 1991; Masson et al., 1994; Hyon et al., 2010). Eye tracking techniques have also been used to detect smooth pursuit tracking of moving stimuli to estimate CS (Mooney et al., 2018).

Preferential-looking (PL) exploits the fact that infants have a greater tendency to fixate on a more interesting or salient stimulus than a plain homogeneous field (Fantz, 1963). The PL technique has been used to measure vision in infants and adults with cognitive or speech impairments (Atkinson et al., 1974; Banks and Salapatek, 1978). For example, the Teller acuity cards (McDonald et al., 1985) have a grating printed on one side and a blank region on the opposite side. By subjectively monitoring the infant’s gaze behavior, an examiner can identify whether the grating can be discriminated from the blank region. A disadvantage of current PL tests is that they require a high level of skill to determine which stimulus the infant fixates on and rely on the subjective judgment of the testing clinician. Previous knowledge about the tested infant’s known or suspected visual disorder could also bias the test results. Eye tracking technology has the potential to remove subjectivity from PL tests and to enable the use of PL without experienced investigators. This study aimed to test the possibility of using an eye-tracker to accurately record eye movements and evaluate the contrast threshold. The study was conducted with a group of healthy adults as a first step toward developing a CS measurement system that combines eye tracking and PL technique for infants and adults with cognitive disabilities.

There are several challenges in designing an integrated eye tracking CS measurement suitable for clinical use. For instance, a fast and precise eye tracking calibration procedure is necessary, and the stimuli have to be positioned appropriately within the visual field for a suitable period of time. In addition, an algorithm for deciding which difficulty level to show on each trial and when to terminate the test is required. An algorithm for identifying gaze position and assessing whether the participant can see the presented optotype is also necessary.

In this study, we developed a novel paradigm to objectively and accurately assess CS using an eye tracking technology to potentially broaden its application on pre-verbal children and others with speech impairments. We used Gabor patches, the standard CS measurement optotype, which were attractive enough for non-verbal children, as the stimuli. Using initial pilot measurements, we determined the appropriate position and duration of stimulus presentation, and the number of trials required for accurate results interpretation. A widely used three-down, one-up staircase procedure that decreased signal contrast by 10% (multiplied the previous value by 0.9) after every three consecutive correct responses and increased signal contrast by 10% after every incorrect response was adopted in estimating contrast thresholds (Huang et al., 2008). Moreover, the five-alternative forced-choice method rather than the traditional two-interval forced-choice method was used in the eye tracking test (elk) which is highly recommended for inexperienced observers, especially in children and within a clinical setting (Jakel and Wichmann, 2006). We anticipated that the fixation points obtained by an eye-tracker would be concentrated in the region of the grating pattern where the observer was able to distinguish the stimuli.

To demonstrate the feasibility of applying today’s eye tracking technology to CS measurement, we investigated the consistency between the contrast thresholds obtained from eye movement measurements compared to those using keypress responses. As a first step toward developing a system for use in infants and adults with cognitive or speech impairments, we tested adult participants with normal vision under two conditions: full refractive correction and lens-induced optical blur to simulate a vision defect.

## Materials and Methods

### Participants

A total of 27 healthy subjects, from 19 to 35 years old (mean = 25.4 ± 2.85 years; 10 females) with normal or corrected-to-normal vision and healthy eyes were recruited from The Optometry Clinic of Zhongshan Ophthalmic Center (Guangzhou, China). Nine participants were randomly selected to participate in a repeated test session to verify the repeatability of the experimental results for the eye tracking test on separate days. Ten subjects also repeated the test with optical defocus. They were under-corrected with plus spherical lenses until their VA dropped to logMAR 0.20. The study protocol was approved by the Ethics Committee of Zhongshan Ophthalmic Center of Sun Yat-sen University and adhered to the tenets of the Declaration of Helsinki. All subjects signed informed consents after they were given written and verbal explanations of the nature and purpose of the study.

### Apparatus

Visual acuity was measured using the Binoptometer 4P (OCULUS, Germany) and it was used again to determine the correction needed to create a VA drop of logMAR 0.20 under the blur stimulus condition. The stimuli were presented at a viewing distance of 60 cm on a gamma-corrected display (ASUS ROG SWIFT PG278QR), with a uniform background luminance of 52.1 ± 1.30 cd/m^2^, resolution of 2,560 × 1,440 pixels, and refresh rate of 165 Hz. A special circuit was used to produce a 14-bit gray-level resolution (Li et al., 2003). Experiments were written in MATLAB (MathWorks Inc. Natick, MA, United States) using elements of the Psychophysics Toolbox (Brainard, 1997). Monocular eye movements were recorded using an Eye-link 1000 Infrared eye tracker (SR Research, Ontario, Canada). Eye movements were streamed to the stimulus computer via a high-speed Ethernet connection and were processed in MATLAB using the Eye-link Toolbox.

### Stimuli

**FIGURE 1:**
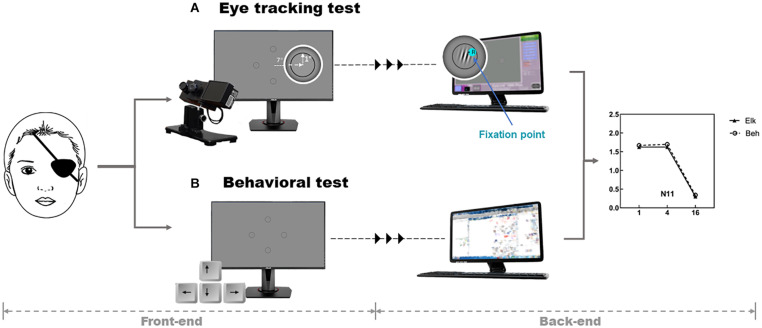
Graphical representation of the study task and procedure. The front-end is the interface on which the subjects performed the test. Monocular CS was estimated via an **(A)** eye tracking test and a conventional psychophysical method [**(B)** Behavioral test], which were both based on a five-alternative forced-choice grating detection task. The task stimuli were the gratings that randomly appeared in one of the four circles, which are equidistant from the tested eye. Each circle had a radius of 1° and were located at the upper, lower, left and right sides, 7° away from the center point of the screen. The observer was instructed to stare at the location where the stimulus appeared and the fixation points recorded by the eye tracker were transmitted to a second monitor. The same task was performed in the Behavioral test using the four arrow keys on a computer keyboard to report perceived location of the stimuli. The examiners observed the subjects’ fixation points and key presses on the second monitor (the back-end) and an automated algorithm presented the next stimulus and determined the contrast threshold. Then, the contrast threshold obtained with two methods were compared across spatial frequencies. elk, eye tracking test; beh, behavioral test (conventional perceptual report).

A sinusoidal grating detection task, in which the test stimuli randomly appeared in one of four circles, as shown in Figure 1, was presented to the participant. The luminance of the four circles was programmed to be homogenous before the test. Michelson contrasts of the sinusoidal gratings were adjusted on each trial by a transformed three-down-one-up staircase (Wetherill and Levitt, 1965) with a proportional step size of 10%, which terminated after 100 trials. Stimulus orientation was varied randomly from 0 to 180 degrees, and the stimuli appeared 25 times in each location. The phase of the Gabor patches was set to zero. The means of the last four staircase reversals were taken as the contrast detection threshold for each spatial frequency (SF) tested [1, 4 and 16 cycle per degree (cpd)].

### Procedure

Two experiments were conducted to measure log contrast sensitivity (logCS) by means of eye tracking and conventional perceptual reporting. The order of measurements and spatial frequency presentations was random. Testing was conducted in a dark room. Subjects’ heads were stabilized using a chin and forehead rest. An adhesive eye patch was used to occlude the untested eye. Participants were tested with their best refractive correction and in the presence of a positive blur lens that reduced their VA to 0.20 (logMAR).

#### Eye Tracking Test (Elk)

Each subject underwent a five-point calibration procedure before starting the test session. During the test, the eye tracker was used to record the fixation point while the observer was instructed to stare at the location where they detected the target (a grating pattern) until a break screen was presented. If they could not see any grating patterns within any of the circles, they were instructed to stare at the central blank area of the screen. Fixation on a potential target location for more than 1 s was regarded as a response. In each trial, the stimulus lasted for no more than 6 s, with a one-second break in between trials to allow participants to blink. A fixation point appeared at the center of the display in between each trial to prompt the subject to refixate at the center in order to avoid any misjudgment of the next eye movement. Contrast thresholds were estimated independently for each SF after 100 trials. A longer break time was provided in between each spatial frequency block to minimize visual fatigue.

#### Conventional Perceptual Report: Behavioral Test (Beh)

In this testing design, observers were instructed to report the perceived location of the stimuli using the four arrow keys on a computer keyboard. Observers were given an option to report “I don’t know,” upon which the response was regarded as incorrect. If the observer did not respond after 6 s, the program automatically treated this stimulus as a wrong answer and skipped to the next trial.

### Data Analysis

Data analyses were performed with SPSS version 25 for Windows (SPSS Inc., IBM, Somers, NY, United States). The normal distribution of data was confirmed using the Shapiro–Wilks test. For the best refractive correction condition, the interaction and main effects of spatial frequency (three frequencies) and measurement type (eye tracking vs. keypress) were tested using a two-way repeated measures ANOVA analysis. The differences in logCS obtained with Elk and Beh at each spatial frequency were further compared with paired *t*-tests (Bonferroni corrected). To assess the relationship between the two measurement types, we computed a Pearson correlation coefficient to indicate the strength and directionality of the linear relationships. The test–retest repeatability of the eye tracking test was determined using a Bland–Altman plot and the Pearson correlation between the first and second logCS measurements. The coefficient of repeatability (CoR) was used to determine the test reliability, where a lower value indicated less variability with repeated measurements; therefore, better reliability. It was calculated by multiplying the within-subject standard deviation values from the test–retest differences (test 2 - test 1) by 1.96. The average difference between test and retest represented the bias.

## Results

### Comparison of Contrast Sensitivity Measured Using Eye Tracking and Perceptual Report

**FIGURE 2:**
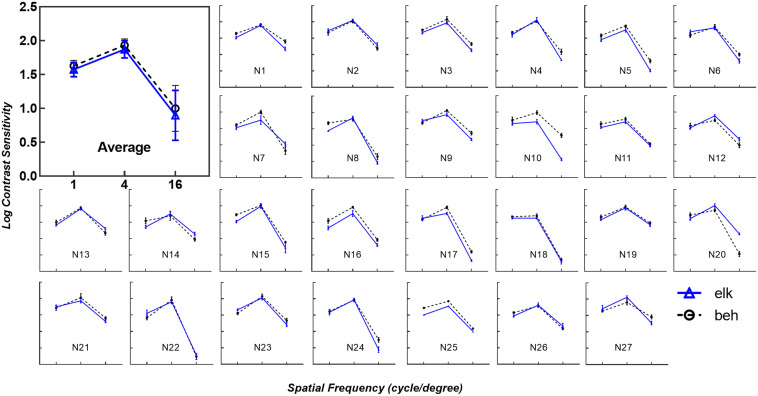
Eye tracking logCS versus behavioral logCS under best-corrected visual acuity (BCVA). The average of all subjects’ data for each spatial frequency is shown in the first subplot (Average). The remaining subplots (N1 to N27) depict the logCS from each subject obtained from the eye tracking test (solid blue line with triangles, elk) and perceptual report (dashed black line with circles, beh) under BCVA. Error bars represent the standard deviation across last four reversals under each spatial frequency. The layout is consistent in each subplot, with the horizontal axes representing spatial frequency in log scale and the vertical axes representing log units of CS. In the average subplot, error bars represent the standard deviation across subjects under each spatial frequency. elk, eye tracking test; beh, behavioral test (conventional perceptual report).

As shown in Figure 2, the contrast thresholds obtained by the eye tracking method (solid blue line) were compared to thresholds measured using the conventional subjective response method (dashed black line). In each subplot, the trend of logCS obtained from the eye tracking method is highly consistent with the conventional CS measure, with the greatest sensitivity at 4 cpd, followed by 1 cpd and 16 cpd. Similar results were noted in the first subplot illustrating the mean logCS of the eye tracking and the behavioral tests based on all participants at each SF.

**FIGURE 3:**
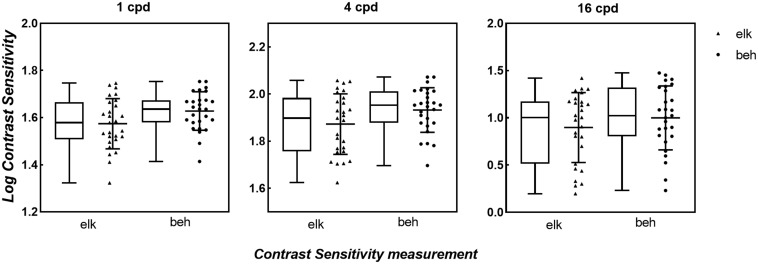
Box and Scatter plots of logCS measured by eye tracking and behavioral tests under BCVA. For each box plot, the middle horizontal lines denote median values of logCS; the top and bottom of each box represent the 25th and 75th percentiles of the data; vertical extending lines and the error bars denote the maximum and the minimum values in each group. For each scatter plot, the middle solid black lines mark the mean of logCS in each SF and the error bars represent ±1 SD. Triangles: logCS of eye tracking method. Circles: logCS of behavioral method. elk, eye tracking test; beh, behavioral test (conventional perceptual report).

**FIGURE 4:**
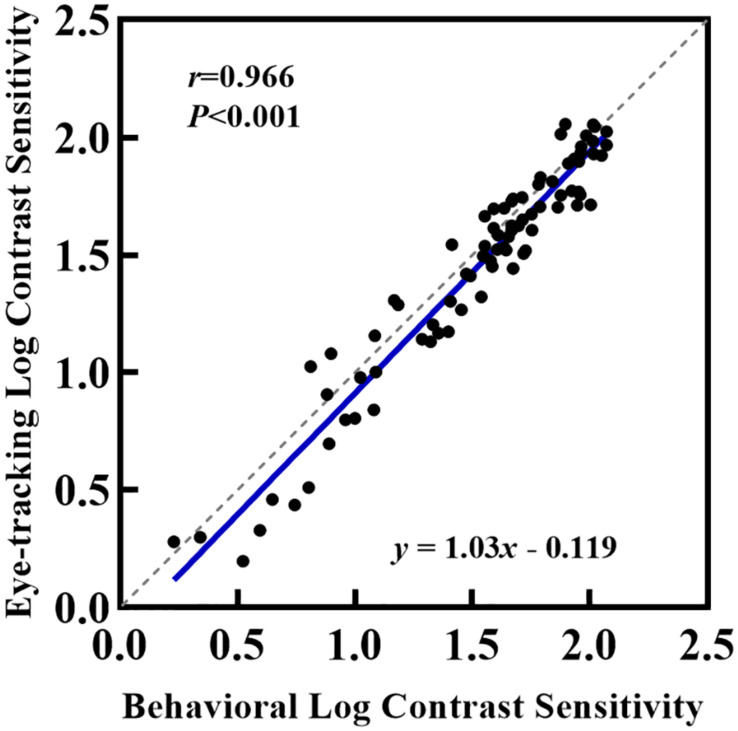
Relationship between logCS estimated from the conventional perceptual and eye tracking report. Each point represents one of the 27 participants who completed eye tracking and behavioral measurements under BCVA. The solid blue line represents the best linear fit to the data.

The box and scatter plots in Figure 3 show the median logCS measured using the eye tracking and the behavioral methods at each SF. A two-way repeated-measures ANOVA was performed on the logCS obtained using the two methods. Mauchly’s sphericity test indicated that for the main effects of measurement type and SF, the assumption of sphericity had been violated, χ^2^(2) = 33.5, *P* < 0.001; therefore, degrees of freedom were corrected using Greenhouse-Geisser estimates of sphericity (ε = 0.575). However, for the measurement type by SF interaction effect, sphericity was met as indicated by, χ^2^(2) = 3.14, *P* = 0.208. ANOVA showed that the measurement type by SF interaction effect was not significant, *F*(2,52.0) = 1.39, *P* = 0.257, while the main effects of both measurement types and SF were significant [*F*_SF_(1,29.9) = 184, *P* < 0.001, η*^2^* = 0.874; *F*_*measurement*_(1,26.0) = 24.1, *P* < 0.001, η*^2^* = 0.481]. Since our study aimed to evaluate the difference between measurements, a paired t-test was then used to compare the logCS obtained with the two measurements at each SF. A statistically significant difference was present between the two methods at each SF [1cpd: *Mean_*elk*_* = 1.57 ± 0.107, *Mean_beh_* = 1.63 ± 0.0810, *Mean_dif_* = 0.0543 ± 0.103, *t*(2,26.0) = 2.72, *P_*corrected*_* = 0.036; 4cpd: *Mean_elk_* = 1.87 ± 0.129, *Mean_*beh*_* = 1.93 ± 0.0952, *Mean_*dif*_* = 0.0601 ± 0.105, *t*(2,26.0) = 2.95, *P_*corrected*_* = 0.003; 16cpd: *Mean_elk_* = 0.897 ± 0.370, *Mean_beh_* = 1.00 ± 0.338, *Mean_dif_* = 0.103 ± 0.152, *t*(2,26.0) = 3.50, *P_*corrected*_* = 0.006]. CS measured using the conventional perceptual reporting method was slightly higher (better) than CS measured using the eye tracking method at each SF. However, linear correlation (blue solid line in Figure 4) shows a strong relationship between logCS measured by the two methods (Pearson’s *r* = 0.966, *P* < 0.001). The linear correlation slope between the two methods was 1.03 [95% confidence interval (CI): 0.969–1.09]. Therefore, we can conclude that the eye tracking technology is comparable to the behavioral method for the measurement of CS.

### Comparison of Contrast Sensitivity Measured Using Eye Tracking and Perceptual Report With Reduced Visual Acuity

**FIGURE 5:**
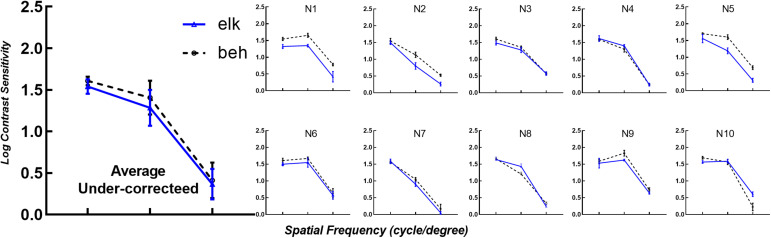
LogCS measured with the two methods with visual acuity reduced to logMAR 0.20. The mean logCS of the eye tracking and behavioral tests of all participants with under-corrected visual acuities for each spatial frequency is shown in the first subplot (Average Under-corrected). The layout and icons are identical to Figure 2. elk, eye tracking test; beh, behavioral test (conventional perceptual report).

To verify the applicability of eye tracking technology for subjects with altered visual functions, we reduced participant’s logMAR VA to 0.20 using optical blur. Data from the eye tracking tests and psychophysical perceptual tests are shown in Figure 5, with identical layout and axes to Figure 2. As shown in Figure 5, the same logCS trend obtained from the two techniques at different spatial frequencies can be observed in each subplot. The logCS of most subjects decreased with increasing spatial frequency.

**FIGURE 6:**
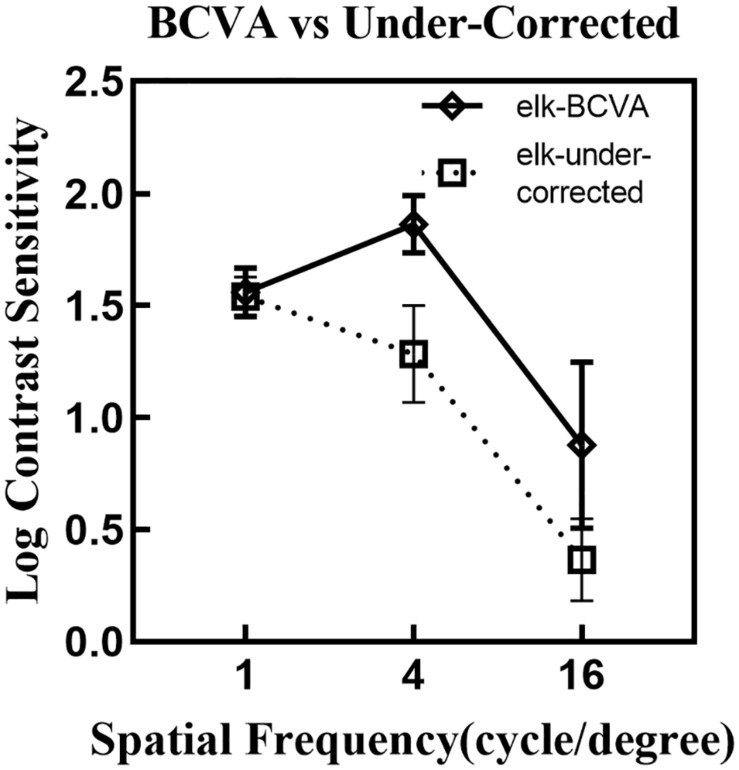
A comparison of the mean logCS at different spatial frequencies under the BCVA (solid line) and under-corrected visual acuity (dotted line) using the eye tracking test. Error bars denote one standard deviation across spatial frequencies. LogCS was significantly reduced at medium and high spatial frequencies, while no distinct difference was noted for the low spatial frequency. BCVA, best-corrected visual acuity; elk, eye tracking test.

Figure 6 compares the logCS at different spatial frequencies under best-corrected VA and under-corrected VA using eye tracking. A drop in logCS is noted when observers were tested in an under-corrected viewing condition.

### Repeatability of the Eye Tracking Test

**FIGURE 7:**
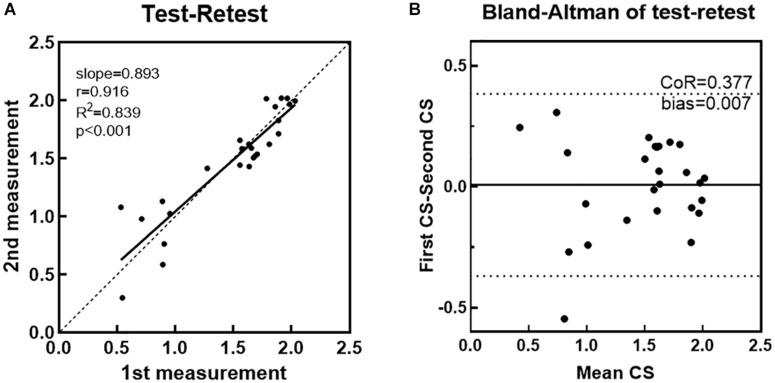
**(A)** LogCS measured on the second run plotted against those obtained in the first run. The Pearson correlation coefficient (r) between two runs was 0.916. Linear regression was performed to evaluate the test–retest reliability. **(B)** The Bland–Altman plot shows the differences (first CS - second CS) between sensitivity estimates obtained from each eye tracking measure, plotted against their mean (first CS + second CS/2). Each dot represents 1 data point. The central solid line indicates the mean difference (bias) between different measures. Dotted lines indicate the 95% agreement upper and lower limit intervals.

Results of the eye tracking test under the same darkroom conditions on different days are illustrated in Figure 7. We calculated the Pearson correlation between logCS measured during the first and second runs, at three different spatial frequencies. The test-retest correlation for the eye tracking method was 0.916, with a slope of 0.893 (95%CI: 0.819–0.962, *R*^2^ = 0.839, Figure 7A). Figure 7B presents the Bland–Altman CoR and bias of eye tracking method. The CoR value was 0.377 logCS (95%CI: −0.369 to 0.384) and the average test–retest difference (bias) was 0.00710 logCS. Both are much lower than those values obtained from the CS test using Gabor patches in previous studies with CoR values ranging from 0.410 to 0.630 logCS and bias ranging from 0.0500 to 0.120 logCS (Thurman et al., 2016). An outlier can be observed in Figure 7B, defined as a test-retest difference exceeding the range expected to contain 95% of the test-retest differences.

## Discussion

Visual fixation is spontaneously attracted to more interesting or salient stimuli. This type of spontaneous behavior is termed PL. As such, some contemporary charts designed to test pre-verbal infant’s vision utilize spatial grating patterns of varying visibility in order to elicit PL, while the examiner observes the infant’s fixation (Brown et al., 2015; Thomas et al., 2021). In this pilot study, we introduced an eye tracker to allow for rapid detection of contrast threshold in adult subjects via a three-down-one-up staircase task starting with low contrast (most difficult level) rather than a trial-by-trial presentation of printed cards. While several challenges in establishing CS measurement using an eye tracker should be addressed, in this study, we proposed point-by-point solutions to challenges encountered, including optimizing calibration, choosing the stimulus, defining the protocol for presenting the stimuli, fixation point judgment, and contrast thresholds.

To verify the feasibility and accuracy of eye tracking technology to test spatial CS, we compared the logCS obtained using the eye tracking format to those from conventional psychophysical perceptual reports. The thresholds measured using perceptual reports were slightly higher than those measured with the eye tracker at each SF. This is most likely caused by fatigue because the eye tracking test usually lasted twice as long as the behavioral test. However, a strong correlation under both best-corrected vision and under-corrected conditions was observed. The logCS results demonstrate sensitivity to an acuity reduction due to under-correction. As Woods and Wood (1995) described, significant loss of CSs at medium and high spatial frequencies were found in refractive blur subjects. Since refractive errors produce an out-of-focus image on the retina, which is equivalent to a low-pass filtered image, the lack of low-frequency components renders them invisible when out of focus (Medina and Howland, 1988). The same results can be observed in the data from our eye tracking tests, which indicate that the eye tracker can precisely record the expected reduction in CS. Moreover, the test–retest repeatability across different days was high. Thus, the eye tracking technology shows a promising application as a reliable objective method for CS assessment within clinical settings.

Our results are in agreement with previous studies that have used eye tracking technology to measure visual function by inducing and quantifying OKN (Schor and Levi, 1980; Leguire et al., 1991; Masson et al., 1994; Hyon et al., 2010) or smooth pursuit tracking of moving stimuli (Mooney et al., 2018). Our work complements these previous studies and provides an alternative paradigm that can be integrated with PL methodology in the future.

However, several cautionary points need to be addressed before the technique can be applied clinically. First, adult subjects were instructed to stare at the location where they detected the target grating, such that, the PL technique was not actually utilized in this pilot study. The feasibility of combining PL technique with an eye tracker for real pre-verbal infants has been previously reported (Vrabič et al., 2021). As such, our subsequent studies will include both pre-verbal infants and pre-literate toddlers in our testing paradigm where we will measure uninstructed response with an eye tracker to test CS. Secondly, it is quite time-consuming as a clinical procedure. In this study, it took subjects approximately 10 min to complete a standard sitting of individual adaptive staircases totaling 100 trials, for each spatial frequency to achieve as many reversals as possible. Visual fatigue associated with prolonged tests resulted in the generally lower logCS with the eye tracking test than those observed with the behavioral test. Moreover, only three SFs were measured for each participant to minimize physical and visual fatigue from distorting the results. Thus, it remains impractical and inadequate to fit the results using a clinical contrast sensitivity function (CSF) plot (Pelli and Bex, 2013). We plan to optimize the testing algorithm by using the quick CSF method. Specifically, the quick CSF uses a computerized Bayesian adaptive framework allowing for direct and quick estimation of several parameters to define a CSF plot (Hou et al., 2010). Such an information-gain testing paradigm is highly efficient and greatly reduces CSF testing time compared with the standard adaptive staircase procedure. Furthermore, the monotonous grating stimuli could be replaced with cartoon patterns filtered with a raised cosine filter and rescaled to different sizes to generate stimuli with different spatial frequencies, so that the paradigm could engage children from different age groups. Lastly, this method might not be suitable for children who are too young to restrain their head movement throughout the testing on a chin rest. A simple and attractive initial calibration interface with free head movement will be more suitable for young children. Although many companies such as Tobii (Tobii Technology) and SMI (SensoMotoric Instruments) offer remote eye tracking (heads free) solutions, they are expensive and remains unsuitable for clinical setting. Sangi et al. (2015) presented offline tools and methods for stabilizing the head based on random facial feature detection to analyze OKN in children. These readily available algorithms and consumer-grade equipment can be adopted in our future optimization model.

This study represents the first step in the development of a paradigm to objectively assess CS via eye-tracking technology, which can be used as a baseline model for a system that can measure visual function in infants and adults with cognitive or speech disabilities. It aimed to develop a testing paradigm that neither depended on observers’ cognitive ability nor their language ability. An eye tracker was employed to judge the observer’s fixation location. Our results from a group of adults with normal vision and their simulated defocused viewing condition provided a preliminary model, which demonstrated the feasibility and accuracy of our method. Strong agreement was found between the measurements made using the eye tracking test and those made using conventional psychophysical perceptual reports.

## Data Availability Statement

The raw data supporting the conclusions of this article will be made available by the authors, without undue reservation.

## Ethics Statement

The studies involving human participants were reviewed and approved by Zhongshan Ophthalmic Center Ethics Committee. The patients/participants provided their written informed consent to participate in this study.

## Author Contributions

JL, JY, YZ, and LG designed the research. YZ, LG, LF, and QY performed the research. YZ and LG analyzed the data and drafted the manuscript. YZ, LG, JC, LC, JL, and JY revised the manuscript. All authors commented on and edited the manuscript, and approved the final version of the manuscript.

## Conflict of Interest

The authors declare that the research was conducted in the absence of any commercial or financial relationships that could be construed as a potential conflict of interest.

## Publisher’s Note

All claims expressed in this article are solely those of the authors and do not necessarily represent those of their affiliated organizations, or those of the publisher, the editors and the reviewers. Any product that may be evaluated in this article, or claim that may be made by its manufacturer, is not guaranteed or endorsed by the publisher.
